# Traffic Load Optimization for Multi-Satellite Relay Systems in Space Information Network: A Proportional Fairness Approach

**DOI:** 10.3390/s22228806

**Published:** 2022-11-14

**Authors:** Xudong Zhong, Baoquan Ren, Xiangwu Gong, Hongjun Li

**Affiliations:** Institute of Systems Engineering, Academy of Military Sciences, People’s Liberation Army (PLA), Beijing 100101, China

**Keywords:** satellite communication, traffic load balancing, radio resource management, fairness resource allocation

## Abstract

Backbone satellites in a space information network (SIN) can be used as air base stations or data relay satellites (DRSs) to realize cross-system, cross-network and long-distance relay transmission. In this paper, a traffic load optimization problem for multi-satellite relay systems in SIN is considered to achieve highly efficient cooperative transmission and improve resource utility. A model of SIN based on a distributed satellite cluster (DSC) is considered, and the characteristics of the model are analyzed. Based on this, a hybrid resource management architecture combining distributed and central resources control schemes is proposed to realize a centrally controllable and distributed optimization of resources to meet various comprehensive service requirements. Two scenarios of multi-satellite relay systems in SIN are given, and traffic load optimization problems with joint bandwidth and power allocation for these two scenarios are formulated based on proportional fairness (PF) criterion to achieve traffic load balancing with considerable system capacity. The optimization problems in these two scenarios are proved to be a convex optimization problem with mathematical analysis, and the closed-form solutions of two problems in their dual domain are derived by dual transformation. With the closed-form solutions, two iterative algorithms based on the subgradient method are designed under the proposed hybrid resource management architecture to solve the problems in this paper. Simulation results show that the proposed schemes can effectively improve the upper bound of system capacity by resource sharing and cooperative relay, and it can balance the traffic load well with guarantees of a reasonable level system capacity compared with existing methods.

## 1. Introduction

With the development of wireless communication technology, various communication platforms have formed their own systems to cope with different communication scenarios and network service requirements. However, the heterogeneous structure of various communication systems and differences in communication methods, transmission medium, protocols and hardware platforms make it difficult to exchange and share information among them. At the same time, due to the very limited space radio resource with the limitation of Shannon limit, the service capacity of various communication systems gradually reaches the bottleneck [1]. In order to integrate different communication platforms, the provided integrated network services between space and earth, user cross-system and cross-platform resource sharing to achieve high rate transmission and further improve the utilization of radio resources, the concept of space information network (SIN) came into being [2,3,4,5].

The backbone network of SIN is served by satellites distributed in space. The primary goal of backbone network construction is to have high stability and cope with physical damage and network failure, which can effectively improve network survivability. In order to obtain a stable and destructible backbone network service, the concept of distributed satellite cluster (DSC) is introduced into SIN to realize the backbone network access of SIN through multi-satellite co-orbit and cooperative transmission [6]. In SIN based on DSC, since the backbone satellites operating on geostationary earth orbit (GEO) can be stably connected with the ground, they cannot only serve as a space base station to provide uplink and downlink information services but also serve as a data relay satellite (DRS) to relay space information (such as the data of observation satellites) to ensure timely data transmission and acquisition. At the same time, due to the global interconnection achieved by the DSC structure, SIN might have ultra-long-distance data transmission, which might need to be relayed through multi-DRS. In the above cases, multi-DRS can cooperate to form a multi-satellite relay system to improve relay transmission efficiency. In this system, due to the constrains of link stability, limited visible communication time and lack of radio resources, it is necessary to optimize the load and transmission resources of multi-channel cooperative relay links to improve the stability, reliability and overall transmission capacity of relay and forwarding.

To the best of our knowledge, the resource optimization problem for a wireless cooperative relay network has not been well investigated. The resource management method related to a wireless cooperative relay network mostly concentrated on terrestrial networks. In [7], an efficient subchannel assignment scheme and a transmission mode selection strategy have been proposed to solve the resource allocation problem in cognitive radio networks with cooperative relays. A joint channel and relay assignment problem has been investigated in [8], which turned out to be NP-hard, and three simple greedy algorithms have been designed to solve the problem in polynomial time. The authors in [9] proposed a hybrid ant colony optimization algorithm to solve the problem of joint resource assignment, relay selection, and bidirectional transmission scheme selection. A power allocation method was proposed in [10] to obtain a near-optimal power allocation strategy for cooperative relay networks. Energy-efficient resource allocation in simultaneous wireless information and power transfer cooperative wireless networks are analyzed in [11]. The authors in [12] studied resource allocation for a wireless-powered relay network, where a hybrid relay with constant energy supply assists an energy-constrained source to send confidential information to a destination. Due to the frequent on–off problem of space links caused by mobility and the difference in transmission media and access mode compared with terrestrial networks, the above work usually cannot be directly applied to the satellite cooperative relay system. In [13], the authors considered the cooperative mechanism of relay satellites deployed in the GEO and low earth orbit (LEO) according to their different transport performances and orbital characteristics. A novel optimization method was proposed in [14] to solve the beam-scheduling problem for the scenario of various mission demands in the DRS system. The resource allocation problem in DRS systems was investigated in [15] from the perspective of joint coordination of users’ selfish behavior in submitting service requests and mission scheduling. In addition, some researchers have completed some enlightening works on inter-satellite routing [16], transmission quality of service (QoS) [17], congestion control [18] and transmission efficiency [19] for multi-DRS relay systems in SIN. However, most of the above studies do not consider the characteristics of the link between satellites, such as on–off frequency and channel asymmetry. At the same time, they only consider the selection of multi-DRS without considering the resource optimization and traffic load balance for multi-DRS cooperative relay. The comparison of existing works for cooperative relay in terrestrial and satellite networks mentioned above is shown in Table 1.

This paper concentrates on the traffic load optimization problem of multi-DRS cooperative relay systems in SIN with considering the load capacity difference of source nodes, variety channel conditions for multi-DRS, limited visible time for source nodes and DRSs and limitation of radio resources. The main contributions of the present paper are summarized as follows:

- According to the definition of SIN, the SIN architecture based on DSC has been constructed as a DSCN model, and its main characteristics are analyzed. On this basis, a hybrid resource management architecture with central-distribution combination is designed to adapt to the multi-latitude, hierarchical and distributed radio resource management under a distributed satellite cluster network (DSCN) model.
- Based on the DSCN model, the mathematical models of two kinds of relay scenarios in SIN are given, and the traffic load optimization problems with joint bandwidth and power allocation in two scenarios are proposed according to proportional fairness (PF) criterion to realize traffic load balancing with proper system capacity guarantees for cooperative multi-DRSs relay in SIN.
- Based on the convex optimization theory, it is proved that the two optimization problems proposed in this paper are convex optimization problems, and the closed-form solutions of the two problems in their dual domain are solved by dual transformation. According to the proposed hybrid resource management architecture, two iterative algorithms based on the subgradient method are designed to find the optimal traffic load balancing solutions.

Through the simulation results, the performances of the proposed algorithm are analyzed. The findings of this paper suggest the following. (a) Multi-DRS cooperative relay can effectively improve system capacity. (b) Enhancing the communication load capacity of the DRS can improve the relay performance. (c) The optimal allocation of bandwidth resource has more influence on the system capacity improvement than that of the power resource, and joint allocation can effectively improve the upper bound of system capacity. (d) The proposed algorithms can balance the traffic load well for multi-DRS with asymmetric channel conditions with guarantees of a reasonable level system capacity compared with existing methods.

The rest of paper is organized as follows. Section 2 gives an SIN model and resource management architecture. In Section 3, the traffic load optimization problems for multi-satellite relay systems in the backbone network of an SIN are formulated. The closed-form solution for the traffic load optimization problems and corresponding resource optimization algorithms are derived and designed in Section 4. Numerical simulation results are provided in Section 5. Section 6 concludes the whole paper.

## 2. SIN and Resource Management Architecture Design

SIN is a comprehensive network which combines different communication platforms and systems to achieve the complex information service integration of deep space, sky and ground. According to [20,21], SIN can be defined by Definition 1.

**Definition** **1.** *SIN is a complex infrastructure that consists of satellites and other nodes (such as space vehicles, base station on ground or on the air, mobile and fixed terminals) in the space, which distribute at different altitudes and carry different payloads such as communication and detection modules. These nodes and satellites construct a comprehensive network by dynamic links between each other, and they achieve the systematic application for space information through the real-time acquisition, transmission and processing of massive data in the space.*

In this section, the SIN model based on DSC and the resource management architecture are described and designed based on Definition 1.

### 2.1. Model of SIN Based on DSC

**Figure 1:**
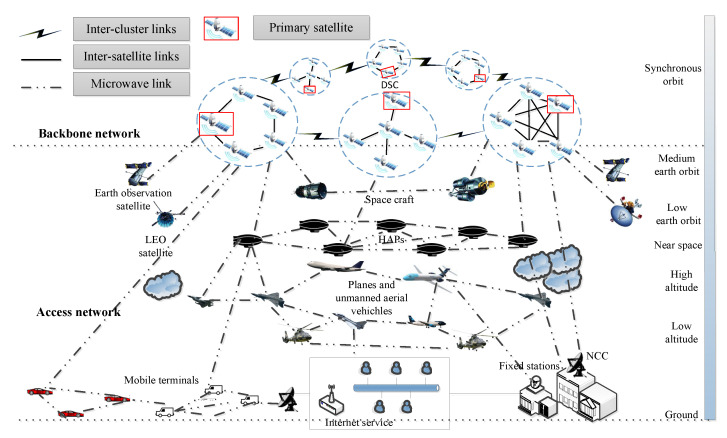
The SIN architecture based on DSC.

Related theories and techniques of SIN are still in progress, and there are no certain models and structure design standards for SIN. Thus, an SIN architecture based on DSC is constructed in this paper according to the model in [21,22] and Definition 1. The architecture is shown in Figure 1.

SIN is divided into two layers from the networking aspect: the access network and the backbone network. The DSC architecture is adopted for the backbone network, and the DSC consists of multiple satellite clusters to construct a distributed satellite network with inter-cluster links (ICLs). Each satellite cluster consists of multiple GEO satellite on the same orbit, and there are inter satellite links (ISLs) connecting satellites to realize different topologies for each cluster with specific function for backbone networking task. Each cluster contains a primary satellite (PS), which realizes the connections with different clusters by ICLs. The ICLs and the ISLs are laser links. The access network is an integrated network with heterogeneous systems and platforms, which includes task and communication platforms distributing on the medium earth orbit and the low earth orbit, in the near space, the high altitude and the low altitude space, or on the ground. These heterogeneous systems and platforms achieve inter-connectivity and integration through the backbone network based on DSC.

In the SIN with a backbone network based on DSC, the systems and the platforms in the access network connect to the backbone network to access data and service; hence, they can be regarded as satellite users for the DSC. Then, the inter-connection between the access network and the backbone network can be described as a DSCN model. According to the payload diversity of the satellites and platforms and the features of links between satellites and users, the characteristics of the DSCN model are described as follows.

- Heterogeneity. The platforms and systems which connect to the access network are heterogeneous from the aspect of logical function structure, system construction, and the communication system and the modulation schemes adopted. The network architectures of the satellites in different clusters are various. Meanwhile, the diversity of the link conditions and the channel states in space and time caused by the characteristic of wide coverage for a satellite, and the network connection by different transmission media (laser and microwave) to provide users with different requirements and various types of services (video, voice, data, etc.), these lead to the height differences of channel conditions and QoS requirements between each access service.
- High dynamic. The topological structure of DSCN changes dynamically with the network demand, network connection condition and channel status. The requests of service resource demand for multiple users are constantly changing, and the resource availability of the entire network is also various at different times.
- The long delay. A GEO satellite is adopted as the backbone satellite in DSCN to provide a stable link for multi-user and multi-system access. Hence, the delay from a GEO satellite to the ground cannot be ignored. At the same time, in the scenario of multi-satellite relay, the routing packets distributed among clusters and satellites cause multi-hop communication from the source satellite to the destination satellite, and such a forwarding mode further increases the network delay.

As can be seen from the above features, the wireless resource management of an SIN is a heterogeneous network resource configuration problem with high complexity due to the distributed characteristics of the network. At the same time, it needs to deal with the impact of network dynamic change and delay. The wireless resource optimization of SIN requires a unified resource management architecture to depict and plan resources, so as to adapt to various features caused by the distributed heterogeneous architecture of SIN. Thus, before the resource optimization of SIN, a reasonable resource management model should be designed according to the network characteristics of DSCN for effective network control.

### 2.2. Resource Management Architecture for DSCN

**Figure 2:**
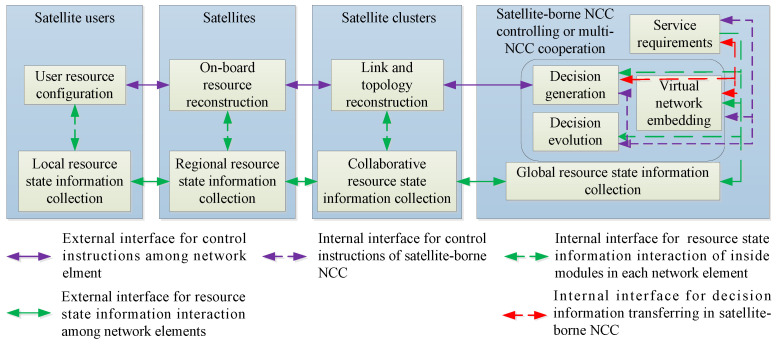
A hybrid resource management architecture for SIN.

In the previous subsection, SIN is described as a DSCN, and the main characteristics of SIN are given. It is necessary to design a reasonable resource management architecture for resource optimization and the management of such a complex comprehensive information service network. In order to adapt to characteristics of heterogeneity, high dynamic and long delay for SIN, and thus realize the rapid discovery and calculation of resources in the whole network, the reconstruction and configuration of local resources, and the central scheduling capability of SIN, SIN needs to have the ability of central control of wireless resources. At the same time, in order to improve the efficiency of resource allocation and reduce the processing delay of wireless resource allocation for some application scenarios, the resource control model of SIN needs to be capable of distributed resource optimization and allocation. In order to realize the centrally controllable and distributed optimization of resources to meet various comprehensive service requirements of SIN, this subsection proposes a hybrid resource management architecture combining distributed and central resources control schemes according to the main characteristics of SIN, which is shown in Figure 2.

As can be seen from Figure 2, the resource state information of the whole network is divided into local resource state information, regional resource state information, collaborative resource state information and global resource state information. A hierarchical structure is formed through user level, satellite level, satellite cluster level and DSCN global level. The resource state information contains parameters such as category, number and availability of resources, and link conditions. Related application protocols and software can be added through software-defined interfaces to realize information sharing and instruction transfer.

In order to shorten the delay of control signaling transmission between the satellites and various platforms accessing to satellites, the main network control functions are carried out by the satellite-borne network control center (NCC), while the ground NCC only uploads necessary update information, codes, data and manual intervention instructions into the satellite through the communication station to achieve network maintenance. The center-distributed hybrid structure is adopted to manage network resources, and each satellite in the backbone network carries a satellite-borne NCC. Data transmission among clusters is achieved by the PS of each cluster, and the primary satellite is the most idle satellite chosen from a cluster. NCCs of multiple PSs can cooperate together to realize collaborative resource management for SIN. Meanwhile, each satellite-borne NCC can also work independently to optimize resource allocation for subnetworks in its coverage area. When collaborative resource management is performed for the global optimization of SIN, satellite users under each backbone satellite sense and collect local resource state information. Through signaling channels, satellite users interact with the backbone satellite, and then, local resource state information is aggregated with resource state information on satellites to form regional resource state information. Regional resource state information is aggregated through ISLs among satellites within the cluster to form the collaborative resource state information of each cluster. The PS of each cluster is in charge of forming global resource state information through ICLs among each other, and they share the information with the whole network as a reference for resource allocation and calculation. Sub-nets of SIN start their own satellite-borne NCC according to the global resources status information (including network demands, capacity, etc.), and distributed computing is adopted to reduce the consumption of resource calculation. The satellite base station on the ground uploads resource allocation algorithms to the satellite-borne NCCs with an interface supplied by virtual network embedding and software definition technology. Then, the NCC calculations generate the respective resource configuration schemes according to the network demands. The resource configuration schemes guide the resource configuration of satellite users, the on-board resource reconstruction of backbone satellites, and the topology reconstruction of cluster links to realize network optimization. Meanwhile, after the resource configuration and reconstruction, the resource configuration and reconstruction results are shared through the internal information interface to realize the update of the resource state information at all levels of SIN. In the meantime, with updating instructions and codes uploaded from ground, the decision generation and calculation algorithms of satellite-borne NCCs can be upgraded, which realize the evolution of global and local decisions for SIN so as to ensure that the network has the ability of dynamic evolution according to the changes of network conditions, user behavior, electromagnetic environment and so on. Through the combination of multi-party distributed computing and central decision making, using virtual network embedding and software-defined technology to achieve different functions, the computing efficiency and flexibility of resource management can be effectively improved. At the same time, each network element in the above architecture can be decoupled and coordinated according to the needs of networking so as to meet the multi-scene and asymmetric resource optimization requirements in SIN.

## 3. System Models and Traffic Load Optimization Problem of Multi-DRS Relay in SIN

In the previous section, some basic concepts of DSC-based SIN have been described briefly, and the main characteristics of SIN have been analyzed. Then, a hybrid resource management architecture is designed based on the central-distributed schemes combination according to these characteristics, and this architecture can supply a solution for the distributed collaboration of multiple satellites and users. Under this architecture, the long-distance data relay of a backbone network can be realized by coordinated multi-satellite transmission. Coordinated multi-satellite transmission is controlled by PSs in each cluster, and multiple backbone satellites can be regarded as DRSs to achieve cooperative data relay with shared transmission resource, which can effectively improve the utilization of resources. In this scenario, in order to improve the relay capacity and avoid the overloading of backbone satellites, it is necessary to optimize the traffic load of an SIN’s backbone network.

### 3.1. Model of Multi-Satellite Relay System

**Figure 3:**
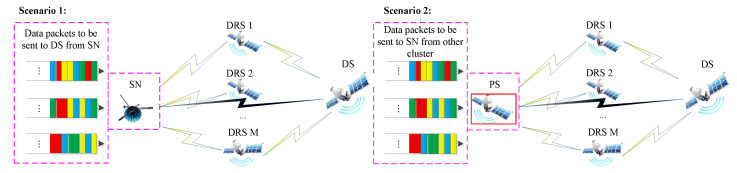
Two multi-satellite relay scenarios for SIN.

In SIN, when there is a link failure or no direct link between the source node and destination node, data transmission can be achieved by multi-satellite relay through a backbone network. Relay transmission through the backbone satellite mainly exists in the following two situations. (1) The destination node (DN) and the source node (SN) are located in the same coverage area of a cluster, and the data are forwarded by the destination satellite (DS) directly covering the destination node. (2) The DN and the SN of the data are located in the coverage areas of different satellite clusters; then, the data can only reach the DS which covers the destination node by crossing multiple clusters through multiple PSs. Therefore, two multi-satellite relay scenarios for SIN are considered in this paper, and their models are shown in Figure 3. For scenario 1, the source node of the data is the communication platform (such as LEO and medium earth orbit (MEO) satellite) operating in non-geosynchronous orbit. This scenario describes a scenario in which non-geostationary communication platforms transmit data packets to the destination node through backbone satellites. For scenario 2, the source node of data is the PS of a cluster, which represents the scenario in which the PS of a cluster receives the data packets sent by PSs of other clusters and forwards them to the destination satellite [23].

In the two scenarios above, data packets of SN can be transmitted to DS by two ways: (1) constructing stable ISLs between SN and DS and (2) constructing relay ISLs to multiple backbone satellites with stable ISLs to DS. Suppose there is a failure or outage of ISL between SN and DS (shown by the gray links in Figure 3); at the same time, there exists *M* backbone satellites which have stable ISLs to DS. Then, *M* backbone satellites can be regarded as DRSs, and packets of SN can be forwarded to DS through these DRS. However, the capacity of a relay channel for one backbone satellite is limited. Hence, in order to improve the efficiency of transmission and reduce the transmission delay, relay data can be forwarded by the cooperative transmission of *M* DRSs. Since ISLs among DRSs are laser links, through satellite orbit position controlling and attitude adjustment, there can be no physical shielding and blocking among satellites. Therefore, the ISL channel for the line-of-sight signal between two satellites can be modeled as a Rician Fading channel with additive white Gaussian noise (AWGN), and the influence of rain attenuation can be ignored. Then, the received signal at time *T* of each DRS can be denoted by [24].

(1) $$y_{s,m,t}=\sqrt{P_{s,m,t}d_{s,m}^{-\gamma }}h_{s,m,t}x_{s,m,t}+n_{s,m,t}$$

where $y_{s,m,t}$ is the received signal of DRS *m* at time *t* from SN *s*, $P_{s,m,t}$ is the transmit power of the signal that SN *s* is sending to DRS *m* at time *t*, $d_{s,m}$ denotes the distance between SN *s* and DRS *m*, γ denotes the path-fading coefficient, $n_{s,m,t}$ denotes the AWGN at time *t* of the ISL between SN *s* and DRS *m*, and $h_{s,m,t}$ is a cyclosymmetry complex Gaussian random variable, which denotes the channel fading coefficient at time *t* of the ISL between SN *s* and DRS *m*. $n_{s,m,t}$ and the channel fading ${\left|h_{s,m,t}\right|}^{2}$ are independently and identically distributed on the ISL between SN *s* and DRS *m*. The mean value and variance of $n_{s,m,t}$ are 0 and $N_{0}$, respectively. The probability density function of ${\left|h_{s,m,t}\right|}^{2}$ is denoted by [24]

(2) $$f_{{\left|h_{s,m,t}\right|}^{2}}\left(h\right)=\frac{1}{\sigma^{2}}\exp\left\{-\frac{s^{2}+h}{\sigma^{2}}\right\}I_{0}\left(2\sqrt{\frac{s^{2}h}{\sigma^{4}}}\right)$$

where $s^{2}=\mu_{1}^{2}+\mu_{2}^{2}$ is the power of the line of sight (LoS) signal, $\sigma^{2}$ is the power of the scattering signal, and $I_{0}\left(•\right)$ is the first kind of zeroth order modified Bessel function.

Hence, the channel-power-gain to noise ratio at time *t* on the ISL from SN *s* to DRS *m* can be expressed as

(3) $$r_{s,m,t}=\frac{{\left|h_{s,m,t}\right|}^{2}d_{s,m}^{-\gamma }}{N_{0}}$$

The received signal of each DRS must be higher than the signal to noise ratio (SNR) threshold of the satellite antenna $th_{m}$ [24]; otherwise, the signal cannot be received and forwarded accurately. Then, we have

(4) $$P_{s,m,t}r_{s,m,t}=\frac{P_{s,m,t}{\left|h_{s,m,t}\right|}^{2}d_{s,m}^{-\gamma }}{N_{0}}\geq th_{m}$$

For scenario 1 in Figure 3, due to the existing of relative motion due to differences in orbital position and operating period, DRSs are periodically visible to SN *s*. Hence, there is a window time for relay, which can be denoted by $T_{a}$. Since the bandwidth of SN in scenario 1 is limited, the bandwidth of ISL between SN and DRS is divided into $T_{a}$ time-slots. Then, we use $\delta_{s,m,t}$ to denote the occupancy of time-slots *t* in the bandwidth of SN *s* for DRS *m*, where $\delta_{s,m,t}=1$ and $\delta_{s,m,t}=0$ where the mean time-slot *t* is and is not occupied by DRS *m*, respectively. Use $C_{s,m}$ to denote the capacity of received relay data from SN *s* for DRS *m* during $T_{a}$, which can be formulated as follows according to the Shannon formula [13,24].

(5) $$C_{s,m}=\frac{1}{T_{a}}\sum_{t=1}^{T_{a}}\delta_{s,m,t}\mathrm{lb}\left(1+P_{s,m,t}r_{s,m,t}\right)$$

As for scenario 2, SN and DRSs are both on stationary orbit; hence, the transmitting time is not limited by the visibility between SN and DRSs. The relay period $T_{b}$ is used to keep the stability of the data queue for SN and avoid congestion of the queue cache caused by a long queue length. The data have not been sent during $T_{b}$ and would be deleted after a relay period; then, the SN as well as PS in the cluster would inform the PS of the cluster where the data came from for retransmission through its ICL. The bandwidth of SN is relatively wider compared to scenario 1; hence, the bandwidth *B* can be divided into multiple sub-bandwidths and allocated as required to improve resource utility. Use $B_{s,m,t}$ to denote the bandwidth allocated by SN *s* to DRS *m* at time-slot *t*; similarly, according to the Shannon formula, the capacity of the signal at DRS *m* received from SN *s* which is denoted by $C_{s,m}$ can be formulated as

(6) $$C_{s,m}=\frac{1}{T_{b}B}\sum_{t=1}^{T_{b}}B_{s,m,t}\mathrm{lb}\left(1+P_{s,m,t}r_{s,m,t}\right)$$

### 3.2. Problem Formulation Based on PF Criterion

Since the main function of the SN in scenario 1 is not communication, the payload is limited and the ability of transmission is relatively low. Hence, a reasonable assumption can be made that the SN in scenario 1 only has one laser antenna; then, the laser antenna can only be aligned with one DRS during a time-slot. Suppose that the antenna adjustment algorithm is running by a specialized software module with an independent calculation unit, which can be accomplished synchronously with signal transmission; then, the time of adjustment can be ignored. To transmit data as much as possible during $T_{a}$, the occupancy of time-slots should be optimized according to the queue length and the link conditions of ISLs between SN and DRSs.

For scenario 2, the SN as well as PS has a powerful payload, and it can be assumed that the number of laser antennas is lager than *M*, which means the SN in scenario 2 can adjust laser antennas to aim at *M* DRSs simultaneously. Similarly, in order to improve the transmission capacity during $T_{b}$, bandwidths allocated to *M* DRSs should be optimized according to the queue length and the link conditions of ISLs between SN and DRSs.

Furthermore, for the above-mentioned two scenarios, the transmit power needs to be adjusted reasonably under different link conditions to satisfy the capacity requirements of each queue and constrains of the receiving SNR threshold for DRSs. Meanwhile, when there comes a bulk data flow, the traffic load at each DRS should be well-balanced to avoid data overload for each DRS, which would cause data congestion or even packet loss. In fact, the traffic load optimization problem can be regarded as a fairness issue for resource allocation, which means fairness resource allocation among multiple DRSs with optimal capacity. In this paper, the PF criterion is adopted to formulate the capacity fairness among DRSs, which can be denoted by [25].

(7) $$\max\sum_{m=1}^{M}\ln\left(U_{m}\right)$$

where $U_{m}$ is the utility function for DRS *m*.

In scenario 1, a time-slot can only allocated to one DRS, which can be denoted by $\sum_{m=1}^{M}\delta_{s,m,t}\leq 1,\;\forall t$. In scenario 2, the bandwidth allocated to one DRS should be no more than the total bandwidth, which can be denoted by $\sum_{m=1}^{M}B_{s,m,t}\leq B,\;\forall t$. For these two scenarios, the total capacity of *M* DRSs cannot exceed the total capacity of the packets that need to be transmitted, which can be denoted by $\frac{1}{T_{a}}\sum_{m=1}^{M}\sum_{t=1}^{T_{a}}\delta_{s,m,t}\mathrm{lb}\left(1+P_{s,m,t}r_{s,m,t}\right)\leq C_{s1}$ and $\frac{1}{T_{b}B}\sum_{m=1}^{M}\sum_{t=1}^{T_{b}}B_{s,m,t}\mathrm{lb}\left(1+P_{s,m,t}r_{s,m,t}\right)\leq C_{s2}$ for scenarios 1 and 2, respectively, where $C_{s1}$ and $C_{s2}$ are the total capacity of packets that need to be transmitted for two scenarios. The transmit power at each time-slot should be no more than the total power, which can be denoted by $P_{s,m,t}\leq P_{1,\mathrm{total}},\;\forall m,t$ and $\sum_{m=1}^{M}P_{s,m,t}\leq P_{2,\mathrm{total}},\;\forall t$, where $P_{1,\mathrm{total}}$ and $P_{2,\mathrm{total}}$ represent the total power in scenario 1 and 2, respectively. In addition, the received signal of each DRS must be higher than the SNR threshold of the satellite antenna $th_{m}$, which is shown in Equation (4).

Let $U_{m}=C_{s,m}$, in order to obtain a fair allocation of bandwidth and power resource, two joint bandwidth (time-slots) and power allocation problems with the constrains mentioned above for traffic load optimization in two scenarios can be formulated as follows.

Scenario 1:

(8) $$\max\sum_{m=1}^{M}\ln\left(\frac{1}{T_{a}}\sum_{t=1}^{T_{a}}\delta_{s,m,t}\mathrm{lb}\left(1+P_{s,m,t}r_{s,m,t}\right)\right)$$

(9) $$\begin{array}{cc} s.t. & C1:\frac{1}{T_{a}}\sum_{m=1}^{M}\sum_{t=1}^{T_{a}}\delta_{s,m,t}\mathrm{lb}\left(1+P_{s,m,t}r_{s,m,t}\right)\leq C_{s1}\\ & C2:P_{s,m,t}r_{s,m,t}\geq th_{m},\;\forall m,t\\ & C3:P_{s,m,t}\leq P_{1,\mathrm{total}},\;\forall m,t\\ & C4:\sum_{m=1}^{M}\delta_{s,m,t}\leq 1,\;\forall t \end{array}$$

C1 is the constrain of total capacity, which ensures that the total capacity of *M* DRSs is not more than $C_{s1}$. C2 denotes the constrain of the receiving SNR threshold, which ensures that the transmit power can satisfy the receiving SNR threshold. C3 is the constrain of total power, which ensures that the transmit power at each time-slot is less than or equal to total power. C4 is the constrain of time-slot occupation, which ensures that one time-slot can only be occupied by one DRS; in other words, the laser antenna of SN can only align with one DRS.

Scenario 2:

(10) $$\max\sum_{m=1}^{M}\ln\left(\frac{1}{T_{b}B}\sum_{t=1}^{T_{b}}B_{s,m,t}\mathrm{lb}\left(1+P_{s,m,t}r_{s,m,t}\right)\right)$$

(11) $$\begin{array}{cc} s.t. & C1:\frac{1}{T_{b}B}\sum_{m=1}^{M}\sum_{t=1}^{T_{b}}B_{s,m,t}\mathrm{lb}\left(1+P_{s,m,t}r_{s,m,t}\right)\leq C_{s2}\\ & C2:P_{s,m,t}r_{s,m,t}\geq th_{m},\;\forall m,t\\ & C3:\sum_{m=1}^{M}P_{s,m,t}\leq P_{2,\mathrm{total}},\;\forall t\\ & C4:\sum_{m=1}^{M}B_{s,m,t}\leq B,\;\forall t \end{array}$$

Similar to scenario 1, C1 is the constrain of total capacity, C2 denotes the constrain of the receiving SNR threshold, C3 is the constrain of total power, and C4 is the constrain of bandwidth occupation, which ensures the summation of allocated bandwidths for DRSs to be not more than the total bandwidth *B*.

## 4. Traffic Load Optimization Algorithm Based on Dual Iteration

In order to solve the above-mentioned two traffic load optimization problems, the mathematical properties should be analyzed. Generally speaking, an optimization problem can be solved by a convex optimal method, while it is or can be transferred into a convex optimization problem. The properties of concave–convex for two objective functions are expressed as shown in Theorem  1.

**Theorem** **1.** *The objective function $f_{1}\left(\delta_{s,m,t},P_{s,m,t}\right)=\frac{1}{T_{a}}\sum_{t=1}^{T_{a}}\delta_{s,m,t}\mathrm{lb}\left(1+P_{s,m,t}r_{s,m,t}\right)$ in scenario 1 and the objective function $f_{2}\left(B_{s,m,t},P_{s,m,t}\right)=\frac{1}{T_{b}B}\sum_{t=1}^{T_{b}}B_{s,m,t}\mathrm{lb}\left(1+P_{s,m,t}r_{s,m,t}\right)$ in scenario 2 are concave functions.*

**Proof** **of** **Theorem 1.** For scenario 1, when $\delta_{s,m,t}=0$, $f_{1}\left(\delta_{s,m,t},P_{s,m,t}\right)=0$. Hence, only the condition of $\delta_{s,m,t}=1$ needs to be considered. Taking the first derivative of $f_{1}\left(1,P_{s,m,t}\right)$ with respect to $P_{s,m,t}$, we have   

(12) $$\frac{\partial f_{1}\left(1,P_{s,m,t}\right)}{\partial P_{s,m,t}}=\frac{r_{s,m,t}}{T_{a}\left(1+P_{s,m,t}r_{s,m,t}\right)\ln2}$$

Obviously, $\frac{\partial f_{1}\left(1,P_{s,m,t}\right)}{\partial P_{s,m,t}}>0$.

The second derivative of $f_{1}\left(1,P_{s,m,t}\right)$ with respect to $P_{s,m,t}$ is denoted by

(13) $$\frac{\partial^{2}f_{1}\left(1,P_{s,m,t}\right)}{\partial {P_{s,m,t}}^{2}}=\frac{r_{s,m,t}^{2}}{T_{a}{\left(1+P_{s,m,t}r_{s,m,t}\right)}^{2}\ln2}$$

which is higher than 0 two. Hence, according to the definition of concave function, $f_{1}\left(\delta_{s,m,t},P_{s,m,t}\right)$ is proved to be concave.

As for scenario 2, let $B_{s,m,t}\mathrm{lb}\left(1+P_{s,m,t}r_{s,m,t}\right)=C_{0}$; then, we have

(14) $$P_{s,m,t}=2^{\frac{C_{0}}{B_{s,m,t}}}-1$$

Plugging Equation (14) into $f_{2}\left(B_{s,m,t},P_{s,m,t}\right)$, we have

(15) $$f_{2}\left(B_{s,m,t},2^{\frac{C_{0}}{B_{s,m,t}}}-1\right)=\frac{1}{T_{b}B}\sum_{t=1}^{T_{b}}B_{s,m,t}\mathrm{lb}\left(1+\left(2^{\frac{C_{0}}{B_{s,m,t}}}-1\right)r_{s,m,t}\right)$$

Taking the first derivative of $f_{2}\left(B_{s,m,t},2^{\frac{C_{0}}{B_{s,m,t}}}-1\right)$ with respect to $B_{s,m,t}$, we have

(16) $$\frac{\partial f_{2}\left(B_{s,m,t},2^{\frac{C_{0}}{B_{s,m,t}}}-1\right)}{\partial B_{s,m,t}}=\frac{1}{B}\mathrm{lb}\left(1+\left(2^{\frac{C_{0}}{B_{s,m,t}}}-1\right)r_{s,m,t}\right)\left(1+\frac{2^{\frac{C_{0}}{B_{s,m,t}}}r_{s,m,t}}{\left(1+\left(2^{\frac{C_{0}}{B_{s,m,t}}}-1\right)r_{s,m,t}\right)\ln2}\right)$$

Through simple mathematical analysis, it can be found that the Equation (16) is higher than 0.

Then, the second derivative of $f_{2}\left(B_{s,m,t},2^{\frac{C_{0}}{B_{s,m,t}}}-1\right)$ with respect to $B_{s,m,t}$ is denoted by

(17) $$\begin{array}{c} \frac{\partial^{2}f_{2}\left(B_{s,m,t},2^{\frac{C_{0}}{B_{s,m,t}}}-1\right)}{\partial {B_{s,m,t}}^{2}}=\frac{1}{B}\frac{2^{\frac{C_{0}}{B_{s,m,t}}}r_{s,m,t}}{\left(1+\left(2^{\frac{C_{0}}{B_{s,m,t}}}-1\right)r_{s,m,t}\right)\ln2}\times \\ \left(1+\frac{1}{\left(1+\left(2^{\frac{C_{0}}{B_{s,m,t}}}-1\right)r_{s,m,t}\right)}\left(\frac{2^{\frac{C_{0}}{B_{s,m,t}}}r_{s,m,t}}{\ln2}+\frac{C_{0}}{B_{s,m,t}^{2}}\mathrm{lb}\left(1+\left(2^{\frac{C_{0}}{B_{s,m,t}}}-1\right)r_{s,m,t}\right)\right)\right) \end{array}$$

It is easy to prove that Equation (17) is higher than 0. Hence, $f_{2}\left(B_{s,m,t},P_{s,m,t}\right)$ is a concave function, too.    □

Meanwhile, it is easy to prove that the solution spaces constructed by the constrains of two problems are convex spaces, and the functions of two problems are the cumulative sums after taking the logarithm of two objective functions. Therefore, two functions of the optimization problems are concave; then, these two problems are convex optimization problems according to convex optimal theory [26], which means the distance between solutions of dual problems and original problems can be regarded as 0 [27], and these two problems can be transferred into dual problems and solved in their dual domain.

### 4.1. Closed-Form Solutions in Scenario 1

The optimization problem in this paper can be solved by minimizing its dual problem. By introducing Lagrange multipliers $\lambda_{1}$, $\left\{\alpha_{1,m,t}\right\}$ and $\left\{\beta_{1,t}\right\}$, the Lagrangian function of the problem in scenario 1 can be denoted by   

(18) $$\begin{array}{c} L_{1}\left(\lambda_{1},\left\{\alpha_{1,m,t}\right\},\left\{\beta_{1,t}\right\},P_{1},\Delta_{1}\right)=\sum_{m=1}^{M}\ln\left(\frac{1}{T_{a}}\sum_{t=1}^{T_{a}}\delta_{s,m,t}\mathrm{lb}\left(1+P_{s,m,t}r_{s,m,t}\right)\right)+\\ \;\;\;\;\;\;\;\;\;\;\;\;\;\;\;\;\;\;\;\;\;\;\;\;\;\;\;\;\;\;\;\lambda_{1}\left(\frac{1}{T_{a}}\sum_{m=1}^{M}\sum_{t=1}^{T_{a}}\delta_{s,m,t}\mathrm{lb}\left(1+P_{s,m,t}r_{s,m,t}\right)-C_{s1}\right)-\\ \;\;\;\;\;\;\;\;\;\;\;\;\;\;\;\;\;\;\;\;\;\;\;\;\;\;\;\;\;\;\;\sum_{m=1}^{M}\sum_{t=1}^{T_{a}}\alpha_{1,m,t}\left(P_{s,m,t}r_{s,m,t}-th_{m}\right)-\sum_{t=1}^{T_{a}}\beta_{1,t}\left(\sum_{m=1}^{M}\delta_{s,m,t}-1\right) \end{array}$$

where $\lambda_{1}\geq 0$, $\alpha_{1,m,t}\geq 0,\;\forall m,t$, and $\beta_{1,t}\geq 0,\;\forall t$. $P_{1}={\left[P_{s,m,t}\right]}_{M\times T_{a}}$ is a power allocation matrix, which denotes the power values of SN *s* in ISLs to *M* DRSs at each time-slot. $\Delta_{1}={\left[\delta_{s,m,t}\right]}_{M\times T_{a}}$ is the time-slot allocation matrix, which denotes time-slots occupation during $T_{a}$. Thus, the dual function is denoted by

(19) $$D_{1}\left(\lambda_{1},\left\{\alpha_{1,m,t}\right\},\left\{\beta_{1,t}\right\}\right)=\max_{P_{1},\Delta_{1}}L_{1}\left(\lambda_{1},\left\{\alpha_{1,m,t}\right\},\left\{\beta_{1,t}\right\},P_{1},\Delta_{1}\right)$$

Hence, the problem can transferred into a dual problem, which can be expressed as

(20) $$\left\{\begin{array}{c} \min_{\lambda_{1},\left\{\alpha_{1,m,t}\right\},\left\{\beta_{1,t}\right\}}D_{1}\left(\lambda_{1},\left\{\alpha_{1,m,t}\right\},\left\{\beta_{1,t}\right\}\right)=\min_{\lambda_{1},\left\{\alpha_{1,m,t}\right\},\left\{\beta_{1,t}\right\}}\max_{P_{1},\Delta_{1}}L_{1}\left(\lambda_{1},\left\{\alpha_{1,m,t}\right\},\left\{\beta_{1,t}\right\},P_{1},\Delta_{1}\right)\\ s.t.\;\lambda_{1}\geq 0;\alpha_{1,m,t}\geq 0,\;\forall m,t;\;\beta_{1,t}\geq 0,\;\forall t;\;P_{s,m,t}\leq P_{1,\mathrm{total}},\;\forall m,t \end{array}\right.$$

By simplifying, decomposing, merging, etc., Equation (18) can be rewritten as

(21) $$L_{1}\left(\lambda_{1},\left\{\alpha_{1,m,t}\right\},\left\{\beta_{1,t}\right\},P_{1},\Delta_{1}\right)=L_{1}^{*}\left(\lambda_{1},\left\{\alpha_{1,m,t}\right\},\left\{\beta_{1,t}\right\},P_{1},\delta_{1}\right)-\lambda_{1}C_{s1}+\sum_{m=1}^{M}\sum_{t=1}^{T_{a}}\alpha_{1,m,t}th_{m}+\sum_{t=1}^{T_{a}}\beta_{1,t}$$

where $L_{1}^{*}\left(\lambda_{1},\left\{\alpha_{1,m,t}\right\},\left\{\beta_{1,t}\right\},P_{1},\delta_{1}\right)$ is the component including $P_{1}$ and $\Delta_{1}$, which can be denoted by

(22) $$\begin{array}{c} L_{1}^{*}\left(\lambda_{1},\left\{\alpha_{1,m,t}\right\},\left\{\beta_{1,t}\right\},P_{1},\Delta_{1}\right)=\sum_{m=1}^{M}\ln\left(\frac{1}{T_{a}}\sum_{t=1}^{T_{a}}\delta_{s,m,t}\mathrm{lb}\left(1+P_{s,m,t}r_{s,m,t}\right)\right)\\ \;\;\;\;\;\;\;\;\;\;\;\;\;\;\;\;\;\;\;\;\;\;\;\;\;\;\;\;\;\;\;+\frac{\lambda_{1}}{T_{a}}\sum_{m=1}^{M}\sum_{t=1}^{T_{a}}\delta_{s,m,t}\mathrm{lb}\left(1+P_{s,m,t}r_{s,m,t}\right)\\ \;\;\;\;\;\;\;\;\;\;\;\;\;\;\;\;\;\;\;\;\;\;\;\;\;\;\;\;\;\;\;-\sum_{m=1}^{M}\sum_{t=1}^{T_{a}}\alpha_{1,m,t}P_{s,m,t}r_{s,m,t}-\sum_{m=1}^{M}\sum_{t=1}^{T_{a}}\beta_{1,t}\delta_{s,m,t} \end{array}$$

In fact, $\max_{P_{1},\Delta_{1}}L_{1}\left(\lambda_{1},\left\{\alpha_{1,m,t}\right\},\left\{\beta_{1,t}\right\},P_{1},\Delta_{1}\right)$ is equivalent to $\max_{P_{1},\Delta_{1}}L_{1}^{*}(\lambda_{1},\left\{\alpha_{1,m,t}\right\},\left\{\beta_{1,t}\right\}$, $P_{1},\Delta_{1})$; hence, we have

(23) $$\min_{\lambda_{1},\left\{\alpha_{1,m,t}\right\},\left\{\beta_{1,t}\right\}}D_{1}\left(\lambda_{1},\left\{\alpha_{1,m,t}\right\},\left\{\beta_{1,t}\right\}\right)≅\min_{\lambda_{1},\left\{\alpha_{1,m,t}\right\},\left\{\beta_{1,t}\right\}}\max_{P_{1},\Delta_{1}}L_{1}^{*}\left(\lambda_{1},\left\{\alpha_{1,m,t}\right\},\left\{\beta_{1,t}\right\},P_{1},\Delta_{1}\right)$$

According to Equation (22), the cumulative sum of components for *M* DRSs and the maximization of the dual function are decoupled; therefore, the above problem can be broken down to *M* subproblems. With given $\lambda_{1}$, $\left\{\alpha_{1,m,t}\right\}$ and $\left\{\beta_{1,t}\right\}$, the first derivative of $L_{1}^{*}\left(\lambda_{1},\left\{\alpha_{1,m,t}\right\},\left\{\beta_{1,t}\right\},P_{1},\Delta_{1}\right)$ with respect to $P_{s,m,t}$ can be denoted by

(24) $$\begin{array}{c} \frac{\partial L_{1}^{*}\left(\lambda_{1},\left\{\alpha_{1,m,t}\right\},\left\{\beta_{1,t}\right\},P_{1},\Delta_{1}\right)}{\partial P_{s,m,t}}=\frac{\delta_{s,m,t}r_{s,m,t}}{\left(\frac{\delta_{s,m,t}}{T_{a}}\mathrm{lb}\left(1+P_{s,m,t}r_{s,m,t}\right)\right)\left(1+P_{s,m,t}r_{s,m,t}\right)\ln2}\\ \;\;\;\;\;\;\;\;\;\;\;\;\;\;\;\;\;\;\;\;\;\;\;\;\;\;\;\;\;\;\;\;\;\;\;\;\;\;\;\;\;\;\;\;\;\;\;+\frac{\lambda_{1}\delta_{s,m,t}}{T_{a}\left(1+P_{s,m,t}r_{s,m,t}\right)\ln2}-\alpha_{1,m,t}r_{s,m,t} \end{array}$$

and the first derivative of $L_{1}^{*}\left(\lambda_{1},\left\{\alpha_{1,m,t}\right\},\left\{\beta_{1,t}\right\},P_{1},\Delta_{1}\right)$ with respect to $\delta_{s,m,t}$ can be denoted by

(25) $$\frac{\partial L_{1}^{*}\left(\lambda_{1},\left\{\alpha_{1,m,t}\right\},\left\{\beta_{1,t}\right\},P_{1},\Delta_{1}\right)}{\partial \delta_{s,m,t}}=\frac{1}{\delta_{s,m,t}}+\frac{\lambda_{1}}{T_{a}}\mathrm{lb}\left(1+P_{s,m,t}r_{s,m,t}\right)-\beta_{1,t}$$

For Equation (24), when $\delta_{s,m,t}=0$, the right-hand side of the formula does not make sense. This is due to the coupling relationship between time-slots and power, when a time-slot is not occupied by one DRS, the power in the ISL between the DRS and SN is 0. Hence, we only consider the condition of $\delta_{s,m,t}=1$, according to the Karush–Kuhn–Tucker (KKT) condition [28]. Let Equation (24) be equal to 0, then, we have

(26) $$\frac{T_{a}r_{s,m,t}+\lambda_{1}\log_{2}\left(1+P_{s,m,t}r_{s,m,t}\right)}{T_{a}\mathrm{lb}\left(1+P_{s,m,t}r_{s,m,t}\right)\left(1+P_{s,m,t}r_{s,m,t}\right)\ln2}=\alpha_{1,m,t}r_{s,m,t}$$

After transposing and combining, we have

(27) $$\left(\alpha_{1,m,t}r_{s,m,t}T_{a}\ln2\left(1+P_{s,m,t}r_{s,m,t}\right)-\lambda_{1}\right)\mathrm{lb}\left(1+P_{s,m,t}r_{s,m,t}\right)=T_{a}r_{s,m,t}$$

Take the exponent of 2 from both sides of Equation (27); then, we have

(28) $${\left(1+P_{s,m,t}r_{s,m,t}\right)}^{\left(\alpha_{1,m,t}r_{s,m,t}T_{a}\ln2\left(1+P_{s,m,t}r_{s,m,t}\right)-\lambda_{1}\right)}=2^{T_{a}r_{s,m,t}}$$

Let $\varphi =1+P_{s,m,t}r_{s,m,t}$; then, the equation above can be rewritten as

(29) $$\varphi^{\left(\alpha_{1,m,t}r_{s,m,t}T_{a}\ln2\varphi -\lambda_{1}\right)}=2^{T_{a}r_{s,m,t}}$$

When the total packet quantity is greater than the capacity, constrain $C_{1}$ in scenario 1 is always true, which means that in this case, $\lambda_{1}$ can be considered as 0. When the total packet quantity is less than the capacity, $C_{s1}$ can be regarded as equal to the total packet quantity. Hence, according to convex optimization theory, $\lambda_{1}$ satisfies the following complementary slackness conditions.

(30) $$\left\{\begin{array}{c} \frac{1}{T_{a}}\sum_{m=1}^{M}\sum_{t=1}^{T_{a}}\delta_{s,m,t}^{*}\mathrm{lb}\left(1+P_{s,m,t}^{*}r_{s,m,t}\right)=C_{s1},\;\lambda_{1}>0\\ \frac{1}{T_{a}}\sum_{m=1}^{M}\sum_{t=1}^{T_{a}}\delta_{s,m,t}^{*}\mathrm{lb}\left(1+P_{s,m,t}^{*}r_{s,m,t}\right)\leq C_{s1},\;\lambda_{1}=0 \end{array}\right.$$

where $P_{s,m,t}^{*}$ and $\delta_{s,m,t}^{*}$ are optimal solutions. Let $\lambda_{1}>0$, if $\delta_{s,m,t}$ is relaxed to a continuous number between 0 and 1, then, when $\frac{\partial L_{1}^{*}\left(\lambda_{1},\left\{\alpha_{1,m,t}\right\},\left\{\beta_{1,t}\right\},P_{1},\Delta_{1}\right)}{\partial \delta_{s,m,t}}=0$, $\delta_{s,m,t}$ reaches the maximum value, which is 1. Hence, according to Equation (25), we have

(31) $$\lambda_{1}=\frac{T_{a}\left(\beta_{1,t}-1\right)}{\mathrm{lb}\left(1+P_{s,m,t}r_{s,m,t}\right)}$$

put it into Equation (29); then, we have

(32) $$\varphi^{\varphi }=2^{\frac{\beta_{1,t}+r_{s,m,t}-1}{\alpha_{1,m,t}r_{s,m,t}\ln2}}$$

According to the formal characteristic of Equation (32), the Lambert-W function can be introduced to simplify the equation, which is denoted by

(33) $$\varphi =\exp\left(W\left(\ln\left(2^{\frac{\beta_{1,t}+r_{s,m,t}-1}{\alpha_{1,m,t}r_{s,m,t}\ln2}}\right)\right)\right)$$

where $W\left(\cdot \right)=\sum_{i=1}^{+\infty }\left({\left(-i\right)}^{i-1}/i!\right){\left(\cdot \right)}^{i}$ is the Lambert-W function. Put $\varphi =1+P_{s,m,t}r_{s,m,t}$ into Equation (33), through basic operations such as transposition, the closed-form solution of optimal power $P_{s,m,t}^{*}$ can be derived. Since $P_{s,m,t}^{*}$ is greater or equal to 0, it can be denoted by   

(34) $$P_{s,m,t}^{*}=\left\{\begin{array}{c} \frac{1}{r_{s,m,t}}{\left(\exp\left(W\left(\ln\left(2^{\frac{\beta_{1,t}+r_{s,m,t}-1}{\alpha_{1,m,t}r_{s,m,t}\ln2}}\right)\right)\right)-1\right)}^{+},\;\;\;\delta_{s,m,t}^{*}=1\\ 0,\;\;\;\;\;\;\;\;\;\;\;\;\;\;\;\;\;\;\;\;\;\;\delta_{s,m,t}^{*}=0 \end{array}\right.$$

where ${\left(x\right)}^{+}=\max\left(0,x\right)$.

Equation (25) decreases with $\delta_{s,m,t}$, and when $\delta_{s,m,t}$ is relaxed to a continuous number between 0 and 1, 1 is the maximum value on the domain of $\delta_{s,m,t}$. Hence, Equation (25) reaches its minimum value at $\delta_{s,m,t}^{*}=1$. Then, the closed-form solution of optimal time-slots allocation indexes $\delta_{s,m,t}^{*}$ can be denoted by

(35) $$\delta_{s,m,t}^{*}=\left\{\begin{array}{c} 1,\;\left(m,t\right)=\arg\min\frac{\lambda_{1}}{T_{a}}\mathrm{lb}\left(1+P_{s,m,t}^{*}r_{s,m,t}\right)-\beta_{1,t}\\ 0,\;\left(m,t\right)\neq \arg\min\frac{\lambda_{1}}{T_{a}}\mathrm{lb}\left(1+P_{s,m,t}^{*}r_{s,m,t}\right)-\beta_{1,t} \end{array}\right.$$

### 4.2. Closed-Form Solutions in Scenario 2

Similar to scenario 1, by introducing Lagrange multipliers $\lambda_{2}$, $\left\{\alpha_{2,m,t}\right\}$, $\left\{\beta_{2,t}\right\}$ and $\left\{\pi_{2,t}\right\}$, the Lagrangian function of the problem in scenario 21 can be denoted by

(36) $$\begin{array}{c} L_{2}\left(\lambda_{2},\left\{\alpha_{2,m,t}\right\},\left\{\beta_{2,t}\right\},\left\{\pi_{2,t}\right\},P_{2},B_{2}\right)=\\ L_{2}^{*}\left(\lambda_{2},\left\{\alpha_{2,m,t}\right\},\left\{\beta_{2,t}\right\},\left\{\pi_{2,t}\right\},P_{2},B_{2}\right)-\lambda_{2}C_{s2}+\sum_{m=1}^{M}\sum_{t=1}^{T_{b}}\alpha_{2,m,t}th_{m}+\sum_{t=1}^{T_{b}}\beta_{2,t}B+\sum_{t=1}^{T_{b}}\pi_{2,t}P_{2,total} \end{array}$$

where $\lambda_{2}\geq 0$, $\alpha_{2,m,t}\geq 0,\;\forall m,t$, $\beta_{2,t}\geq 0,\;\forall t$ and $\pi_{2,t}\geq 0,\;\forall t$. $L_{2}^{*}\left(\lambda_{2},\left\{\alpha_{2,m,t}\right\},\left\{\beta_{2,t}\right\},\left\{\pi_{2,t}\right\},P_{2},B_{2}\right)$ is the component including $P_{2}$ and $B_{2}$, which can be denoted by

(37) $$\begin{array}{c} L_{2}^{*}\left(\lambda_{2},\left\{\alpha_{2,m,t}\right\},\left\{\beta_{2,t}\right\},\left\{\pi_{2,t}\right\},P_{2},B_{2}\right)=\sum_{m=1}^{M}\ln\left(\frac{1}{T_{b}B}\sum_{t=1}^{T_{b}}B_{s.m,t}\mathrm{lb}\left(1+P_{s,m,t}r_{s,m,t}\right)\right)+\\ \;\;\;\;\;\;\;\;\;\;\;\;\;\;\;\;\;\;\;\;\;\;\;\;\;\;\;\;\;\;\;\;\;\;\;\;\;\;\;\;\;\;\;\;\;\;\;\;\;\;\;\;\;\;\;\;\;\;\;\;\;\;\;\;\frac{\lambda_{2}}{T_{b}B}\sum_{m=1}^{M}\sum_{t=1}^{T_{b}}B_{s.m,t}\mathrm{lb}\left(1+P_{s,m,t}r_{s,m,t}\right)-\\ \;\;\;\;\;\;\;\;\;\;\;\;\;\;\;\;\;\;\;\;\;\;\;\;\;\;\;\;\;\;\;\;\;\;\;\;\;\;\;\;\;\;\;\;\;\;\;\;\;\;\;\;\;\;\;\;\;\;\;\;\;\;\;\;\sum_{m=1}^{M}\sum_{t=1}^{T_{b}}\alpha_{2,m,t}P_{s,m,t}r_{s,m,t}-\sum_{m=1}^{M}\sum_{t=1}^{T_{b}}\beta_{2,t}B_{s.m,t}-\\ \;\;\;\;\;\;\;\;\;\;\;\;\;\;\;\;\;\;\;\;\;\;\;\;\;\;\;\;\;\;\;\;\;\;\;\;\;\;\;\;\;\;\;\;\;\;\;\;\;\;\;\;\;\;\;\;\;\;\;\;\;\;\;\;\sum_{m=1}^{M}\sum_{t=1}^{T_{b}}\pi_{2,t}P_{s,m,t} \end{array}$$

where $P_{2}={\left[P_{s,m,t}\right]}_{M\times T_{b}}$ is the matrix of power allocation, and $B_{2}={\left[B_{s,m,t}\right]}_{M\times T_{b}}$ is the matrix of bandwidth allocation, which denote the power and bandwidth allocated in each time-slot of ISLs between SN and *M* DRSs, respectively. Then, the dual function can be formulated as

(38) $$D_{2}\left(\lambda_{2},\left\{\alpha_{2,m,t}\right\},\left\{\beta_{2,t}\right\},\left\{\pi_{2,t}\right\}\right)=\max_{P_{2},B_{2}}L_{2}\left(\lambda_{2},\left\{\alpha_{2,m,t}\right\},\left\{\beta_{2,t}\right\},\left\{\pi_{2,t}\right\},P_{2},B_{2}\right)$$

Hence, the original problem can be transferred into a dual problem as follows.

(39) $$\left\{\begin{array}{c} \begin{array}{c} \min_{\lambda_{2},\left\{\alpha_{2,m,t}\right\},\left\{\beta_{2,t}\right\},\left\{\pi_{2,t}\right\}}D_{2}\left(\lambda_{2},\left\{\alpha_{2,m,t}\right\},\left\{\beta_{2,t}\right\},\left\{\pi_{2,t}\right\}\right)=\\ \min_{\lambda_{2},\left\{\alpha_{2,m,t}\right\},\left\{\beta_{2,t}\right\},\left\{\pi_{2,t}\right\}}\max_{P_{2},B_{2}}L_{2}^{*}\left(\lambda_{2},\left\{\alpha_{2,m,t}\right\},\left\{\beta_{2,t}\right\},\left\{\pi_{2,t}\right\},P_{2},B_{2}\right) \end{array}\\ s.t.\;\lambda_{2}\geq 0;\;\alpha_{2,m,t}\geq 0,\;\forall m,t;\;\beta_{2,t}\geq 0,\;\forall t;\;\pi_{2,t}\geq 0,\;\forall t \end{array}\right.$$

According to Equation (37), the dual problem in Equation (39) can be decoupled into *M* subproblems. Similar to the last subsection, with given $\lambda_{2}$, $\left\{\alpha_{2,m,t}\right\}$, $\left\{\beta_{2,t}\right\}$ and $\left\{\pi_{2,t}\right\}$, let $\frac{\partial L_{2}^{*}\left(\lambda_{2},\left\{\alpha_{2,m,t}\right\},\left\{\beta_{2,t}\right\},\left\{\pi_{2,t}\right\},P_{2},B_{2}\right)}{\partial P_{s,m,t}}=0$ and $\frac{\partial L_{2}^{*}\left(\lambda_{2},\left\{\alpha_{2,m,t}\right\},\left\{\beta_{2,t}\right\},\left\{\pi_{2,t}\right\},P_{2},B_{2}\right)}{\partial B_{s,m,t}}=0$. Then, Equations (40) and (41) can be obtained according to the KKT condition.

(40) $$\frac{T_{b}Br_{s,m,t}+\lambda_{2}B_{s,m,t}\ln\left(1+P_{s,m,t}r_{s,m,t}\right)}{T_{b}B\mathrm{lb}\left(1+P_{s,m,t}r_{s,m,t}\right)\left(1+P_{s,m,t}r_{s,m,t}\right)\ln2}=\alpha_{2,m,t}r_{s,m,t}+\pi_{2,t}$$

(41) $$\frac{1}{B_{s,m,t}}=\beta_{2,t}-\frac{\lambda_{2}}{T_{b}B}\ln\left(1+P_{s,m,t}r_{s,m,t}\right)$$

With the transposition of Equation (41), the optimal bandwidth allocation solutions $B_{s,m,t}^{*}$ with given optimal power allocation solutions $P_{s,m,t}^{*}$ can be denoted by

(42) $$B_{s,m,t}^{*}={\left(\frac{T_{b}B}{\left(T_{b}\beta_{2,t}-\lambda_{2}\right)\ln\left(1+P_{s,m,t}^{*}r_{s,m,t}\right)}\right)}^{+}$$

Substituting Equation (42) into Equation (40), we have

(43) $$\frac{T_{b}\beta_{2,t}r_{s,m,t}-\lambda_{2}r_{s,m,t}+\lambda_{2}}{\left(T_{b}\beta_{2,t}-\lambda_{2}\right)\ln\left(1+P_{s,m,t}r_{s,m,t}\right)\left(1+P_{s,m,t}r_{s,m,t}\right)\ln2}=\alpha_{2,m,t}r_{s,m,t}+\pi_{2,t}$$

With the transposition of Equation (43), it can be rewritten as

(44) $$\ln\left(1+P_{s,m,t}r_{s,m,t}\right)\left(1+P_{s,m,t}r_{s,m,t}\right)=\frac{T_{b}\beta_{2,t}r_{s,m,t}-\lambda_{2}r_{s,m,t}+\lambda_{2}}{\left(T_{b}\beta_{2,t}-\lambda_{2}\right)\ln2\left(\alpha_{2,m,t}r_{s,m,t}+\pi_{2,t}\right)}$$

Take the exponent of 2 to both sides of Equation (44); then, we have

(45) $$\frac{T_{b}\beta_{2,t}r_{s,m,t}-\lambda_{2}r_{s,m,t}+\lambda_{2}}{\left(T_{b}\beta_{2,t}-\lambda_{2}\right)\ln\left(1+P_{s,m,t}r_{s,m,t}\right)\left(1+P_{s,m,t}r_{s,m,t}\right)\ln2}=\alpha_{2,m,t}r_{s,m,t}+\pi_{2,t}$$

Similar to the last subsection, by applying the Lambert-W function within Equation (45), the optimal power allocation solutions $P_{s,m,t}^{*}$ can be expressed as follows.

(46) $$P_{s,m,t}^{*}=\left\{\begin{array}{c} \frac{1}{r_{s,m,t}}{\left(\exp\left(W\left(\ln\left(2^{\frac{T_{b}\beta_{2,t}r_{s,m,t}-\lambda_{2}r_{s,m,t}+\lambda_{2}}{\left(T_{b}\beta_{2,t}-\lambda_{2}\right)\ln2\left(\alpha_{2,m,t}r_{s,m,t}+\pi_{2,t}\right)}}\right)\right)\right)-1\right)}^{+},\;\;B_{s,m,t}^{*}\neq 0\\ 0\;\;\;\;\;\;\;\;\;\;\;\;\;\;\;\;\;\;\;\;\;\;\;,\;\;B_{s,m,t}^{*}=0 \end{array}\right.$$

### 4.3. Dual Iteration Optimization Algorithm Based on PF

Through the previous analysis and derivation, we obtain the closed-form solutions of optimal power and bandwidth (time-slot) allocation in two multi-satellite relay scenarios for SIN. However, to solve the two optimization problems in this paper, the optimal Lagrange multipliers must be obtained, which can be solved by iteration methods such as the ellipsoid method and gradient method. In this paper, the gradient method is adopted.

According to the gradient method, the subgradients of Lagrange multipliers in two optimal problems can be expressed by Lemmas 1 and 2, respectively.

**Lemma** **1.** *In scenario 1, the subgradients of $\lambda_{1}$, $\left\{\alpha_{1,m,t}\right\}$ and $\left\{\beta_{1,t}\right\}$ can be, respectively, denoted by*

(47) $$\Delta \lambda_{1}=C_{s1}-\frac{1}{T_{a}}\sum_{m=1}^{M}\sum_{t=1}^{T_{a}}\delta_{s,m,t}\ln\left(1+P_{s,m,t}r_{s,m,t}\right)$$

(48) $$\Delta \beta_{1,t}=1-\sum_{m=1}^{M}\delta_{s,m,t}$$

(49) $$\Delta \beta_{1,t}=1-\sum_{m=1}^{M}\delta_{s,m,t}$$

**Proof** **of** **Lemma 1.** According to Equation (19), we have

(50) $$D\left({\lambda^{\prime }}_{1},\left\{{\alpha^{\prime }}_{1,m,t}\right\},\left\{{\beta^{\prime }}_{1,t}\right\}\right)=\max_{P_{1},\delta_{1}}L\left(P_{1},\Delta_{1},{\lambda^{\prime }}_{1},\left\{{\alpha^{\prime }}_{1,m,t}\right\},\left\{{\beta^{\prime }}_{1,t}\right\}\right)$$

where ${\lambda^{\prime }}_{1}$, $\left\{{\alpha^{\prime }}_{1,m,t}\right\}$ and $\left\{{\beta^{\prime }}_{1,t}\right\}$ are Lagrange multipliers after several times updating with subgradients.

Let $P_{1}^{*}$ and $\Delta_{1}^{*}$ be optimal solutions for $\max_{P_{1},\Delta_{1}}L\left(P_{1},\Delta_{1},\lambda_{1},\left\{\alpha_{1,m,t}\right\},\left\{\beta_{1,t}\right\}\right)$; then, we have

(51) $$D\left({\lambda^{\prime }}_{1},\left\{{\alpha^{\prime }}_{1,m,t}\right\},{\left\{{\beta^{\prime }}_{1,t}\right\}}_{1}\right)\geq \max_{P_{1},\Delta_{1}}L\left(P_{1}^{*},\Delta_{1}^{*},{\lambda^{\prime }}_{1},\left\{{\alpha^{\prime }}_{1,m,t}\right\},\left\{{\beta^{\prime }}_{1,t}\right\}\right)$$

where $L\left(P_{1}^{*},\Delta_{1}^{*},{\lambda^{\prime }}_{1},\left\{{\alpha^{\prime }}_{1,m,t}\right\},\left\{{\beta^{\prime }}_{1,t}\right\}\right)$ is denoted by

(52) $$\begin{array}{c} \begin{array}{c} L\left(P_{1}^{*},\Delta_{1}^{*},{\lambda^{\prime }}_{1},\left\{{\alpha^{\prime }}_{1,m,t}\right\},\left\{{\beta^{\prime }}_{1,t}\right\}\right)=\\ \left({\lambda^{\prime }}_{1}-\lambda_{1}\right)\left(C_{s1}-\frac{1}{T_{a}}\sum_{m=1}^{M}\sum_{t=1}^{T_{a}}\delta_{s,m,t}\ln\left(1+P_{s,m,t}r_{s,m,t}\right)\right)+ \end{array}\\ \sum_{m=1}^{M}\sum_{t=1}^{T_{a}}\left({\alpha^{\prime }}_{1,m,t}-\alpha_{1,m,t}\right)\left(P_{s,m,t}r_{s,m,t}-th_{m}\right)+\\ \sum_{t=1}^{T_{a}}\left({\beta^{\prime }}_{1,t}-\beta_{1,t}\right)\left(1-\sum_{m=1}^{M}\delta_{s,m,t}\right)+L\left(P_{1}^{*},\Delta_{1}^{*},\lambda_{1},\left\{\alpha_{1,m,t}\right\},\left\{\beta_{1,t}\right\}\right) \end{array}$$

Taking the maximum of both sides of this equation, we have

(53) $$\begin{array}{c} D\left({\lambda^{\prime }}_{1},\left\{{\alpha^{\prime }}_{1,m,t}\right\},\left\{{\beta^{\prime }}_{1,t}\right\}\right)\geq \left({\lambda^{\prime }}_{1}-\lambda_{1}\right)\left(C_{s1}-\frac{1}{T_{a}}\sum_{m=1}^{M}\sum_{t=1}^{T_{a}}\delta_{s,m,t}\ln\left(1+P_{s,m,t}r_{s,m,t}\right)\right)+\\ \;\;\;\;\;\;\;\;\;\;\;\;\;\;\;\;\;\;\;\;\;\;\;\;\;\;\;\;\;\;\;\;\;\;\;\;\;\;\;\;\;\;\;\;\;\;\sum_{m=1}^{M}\sum_{t=1}^{T_{a}}\left({\alpha^{\prime }}_{1,m,t}-\alpha_{1,m,t}\right)\left(P_{s,m,t}r_{s,m,t}-th_{m}\right)+\\ \;\;\;\;\;\;\;\;\;\;\;\;\;\;\;\;\;\;\;\;\;\;\;\;\;\;\;\;\;\;\;\;\;\;\;\;\;\;\;\;\;\;\;\;\;\;\sum_{t=1}^{T_{a}}\left({\beta^{\prime }}_{1,t}-\beta_{1,t}\right)\left(1-\sum_{m=1}^{M}\delta_{s,m,t}\right)+D\left(\lambda_{1},\left\{\alpha_{1,m,t}\right\},\left\{\beta_{1,t}\right\}\right) \end{array}$$

which satisfies the definition of a subgradient.    □

**Lemma** **2.** *In scenario 2, the subgradients of $\lambda_{2}$, $\left\{\alpha_{2,m,t}\right\}$, $\left\{\beta_{2,t}\right\}$ and $\left\{\pi_{2,t}\right\}$ can be, respectively, denoted by*

(54) $$\Delta \lambda_{2}=C_{s2}-\frac{1}{T_{b}B}\sum_{m=1}^{M}\sum_{t=1}^{T_{b}}B_{s,m,t}\ln\left(1+P_{s,m,t}r_{s,m,t}\right)$$

(55) $$\Delta \alpha_{2,m,t}=P_{s,m,t}r_{s,m,t}-th_{m}$$

(56) $$\Delta \beta_{2,t}=B-\sum_{m=1}^{M}B_{s,m,t}$$

(57) $$\Delta \pi_{2,t}=P_{2,\mathrm{total}}-\sum_{m=1}^{M}P_{s,m,t}$$

**Proof** **of** **Lemma 2.** The proof is similar to the proof of Lemma 1; hence, the proof process is omitted.    □

Based on subgradients given by Lemmas 1 and 2, Lagrange multipliers can be updated by multi-step iteration. The Lagrange multiplier’s updating methods for two scenarios are shown as follows.

(58) $$\left(\lambda_{j}^{\left(i+1\right)},\alpha_{j,m,t}^{\left(i+1\right)},\beta_{j,t}^{\left(i+1\right)}\right)=\left(\lambda_{j}^{\left(i\right)},\alpha_{j,m,t}^{\left(i\right)},\beta_{j,t}^{\left(i\right)}\right)-{\theta_{1}}^{\left(i\right)}\left(\Delta \lambda_{j},\Delta \alpha_{j,m,t},\Delta \beta_{j,t}\right)$$

(59) $$\left(\lambda_{2}^{\left(i+1\right)},\alpha_{2,m,t}^{\left(i+1\right)},\beta_{2,t}^{\left(i+1\right)},\pi_{2,t}^{\left(i+1\right)}\right)=\left(\lambda_{2}^{\left(i\right)},\alpha_{2,m,t}^{\left(i\right)},\beta_{2,t}^{\left(i\right)},\pi_{2,t}^{\left(i\right)}\right)-{\theta_{2}}^{\left(i\right)}\left(\Delta \lambda_{2},\Delta \alpha_{2,m,t},\Delta \beta_{2,t},\Delta \pi_{2,t}\right)$$

where ${\theta_{j}}^{\left(i\right)}$ is the step length for round *i* iteration for scenario *j*, and the step length must satisfy the following condition.

(60) $$\sum_{i=1}^{\infty }{\theta_{j}}^{\left(i\right)}=\infty ,\lim_{i\to \infty }{\theta_{j}}^{\left(i\right)}=0,j=1,2$$

Based on the proposed resource management architecture, two traffic load optimization algorithms are designed according to Lemmas 1 and 2 and Equations (58) and (59), which are shown in Algorithms 1 and 2. In scenario 1, due to the long distance and the limited visible window time between the SN and the backbone satellite, after the PS of the DS’s cluster selects DRSs to accomplish the relay task, the channel condition parameters during the visible window time are estimated by DRSs. Then, the parameters will be sent to the SN, and the optimal solutions will be calculated by the SN, which can be regarded as a centralized resource management. In scenario 2, the PS is considered as a SN, and the distance between the PS and other satellites in the cluster is relatively close. Hence, the channel condition parameters are estimated within each time-slot by PS to improve the accuracy of channel estimation. Resource calculation is accomplished by DRSs, and PS is in charge of updating Lagrange multipliers. The optimization process in scenario 2 is a distributed resource management with the cooperation of a central node. Obviously, the resource management architecture proposed in this paper can adapt to different communication requirements and effectively improve resource computational efficiency.

**Algorithm 1** Traffic load optimization algorithm in scenario 1.**Input:** Maximum iteration time $I_{\max}$, termination value of iteration ε

**Output:** Optimal transmitting power $P_{1}^{*}$, Optimal time-slot allocation $\Delta_{1}$
 1:SN sends a relay request to a backbone satellite which could be connected within the DS’s cluster, and the backbone satellite transmits the request to PS; 2:Based on collaborative resource state information, PS chooses *M* as a relatively idle satellite, whose links between DS are stable or can be activated for stable connection, as DRSs; 3:PS estimates the communication time $T_{a}$, calculates the number of the time-slots which can be allocated and initializes $\lambda_{1}$, $\left\{\alpha_{1,m,t}\right\}$, $\left\{\beta_{1,t}\right\}$, $P_{1}$ and $\Delta_{1}$; then, it sends these parameters to *M* DRSs; 4:*M* DRSs estimate the channel condition parameters $r_{s,m,t}$ based on regional resource state information, then, they feed $r_{s,m,t}$, $T_{a}$, $\lambda_{1}$, $\left\{\alpha_{1,m,t}\right\}$, $\left\{\beta_{1,t}\right\}$, $P_{1}$ and $\Delta_{1}$ back to SN and inform it the relay request is authorized; 5:SN sets the iteration counter *i*, DRS counter *m* and time-slot counter *t* as 0; 6:**repeat** 7:   i=i+1; 8:   **repeat** 9:       m=m+1; 10:     **repeat** 11:        t=t+1; 12:        SN calculates $P_{s,m,t}$ based on Equation (34); 13:        SN calculates $\delta_{s,m,t}$ based on Equation (35); 14:     **until** $t=T_{b}$ 15:   **until** m=M 16:   SN updates 3 Lagrange multipliers based on Equation (58); 17:**until**$i=I_{\max}$ or $\left(\lambda_{j}^{\left(i\right)},\alpha_{j,m,t}^{\left(i\right)},\beta_{j,t}^{\left(i\right)}\right)\cdot \left(\Delta \lambda_{j},\Delta \alpha_{j,m,t},\Delta \beta_{j,t}\right)\leq \left(\varepsilon ,\varepsilon ,\varepsilon \right)$ 18:SN obtains optimal transmitting power $P_{1}^{*}$ and optimal time-slot allocation $\Delta_{1}$; then, it begins to relay transmission based on the optimization results; 19:In the end of each time-slot, DRSs send an ACK message to SN if the data package is successfully received; then, SN will delete these data from the cache queue; otherwise, the data will be stored in the cache for transmitting during the next visible window time.


ε in two algorithms represents the termination value of the iteration, which is assumed to be the same for each Lagrange multiplier. Lagrange multipliers are initialized by random function, and the initial power values in scenario 1 are equal to the maximum power value ($P_{1,\mathrm{total}}$) for each time-slot, while in scenario 2, the power values are initialized by uniform distribution among DRSs ($P_{2,\mathrm{total}}/M$). $\Delta_{1}$ and $B_{2}$ are initialized by Equations (35) and (42).

**Algorithm 2** Traffic load optimization algorithm in scenario 2.**Input:**
Maximum iteration time $I_{\max}$, termination value of iteration ε

**Output:**
Optimal transmitting power $P_{2}^{*}$, optimal bandwidth allocation $B_{2}^{*}$
 1:Based on collaborative resource state information, PS chooses *M* as a relatively idle satellite whose links with DS are stable or can be activated for stable connection, as DRSs; 2:PS initializes $\lambda_{2}$, $\left\{\alpha_{2,m,t}\right\}$, $\left\{\beta_{2,t}\right\}$, $\left\{\pi_{2,t}\right\}$, $P_{2}$ and $B_{2}$, sets iteration counter *i*, DRS counter *m* and time-slot counter *t* as 0; 3:**repeat** 4:   i=i+1; 5:   **repeat** 6:     m=m+1; 7:     **repeat** 8:           t=t+1; 9:        PS estimates the channel condition parameters based on collaborative resource state information; then, it sends the parameters with $\lambda_{2}$, $\left\{\alpha_{2,m,t}\right\}$, $\left\{\beta_{2,t}\right\}$, $\left\{\pi_{2,t}\right\}$, $P_{2}$ and $B_{2}$ to DRSs. 10:        SN calculates $B_{s,m,t}$ based on Equation (41) and reports it to PS; 11:        SN calculates $P_{s,m,t}$ based on Equation (45) and reports it to PS; 12:     **until** $t=T_{b}$ 13:   **until** m=M 14:   PS updates 4 Lagrange multipliers based on Equation (59); 15:**until**$i=I_{\max}$ or $\left(\lambda_{j}^{\left(i\right)},\alpha_{j,m,t}^{\left(i\right)},\beta_{j,t}^{\left(i\right)},\pi_{j,t}^{\left(i\right)}\right)\cdot \left(\Delta \lambda_{j},\Delta \alpha_{j,m,t},\Delta \beta_{j,t},\Delta \pi_{j,t}\right)\leq \left(\varepsilon ,\varepsilon ,\varepsilon ,\varepsilon \right)$ 16:PR obtains $P_{2}^{*}$ and $B_{2}^{*}$, and it begins relay transmission; 17:At the end of each time-slot, DRSs sends an ACK message to PS if the data package is successfully received; then, PS will delete these data from the cache queue; otherwise, the data will be stored in the cache for transmitting during the next relay period.


## 5. Simulation Results and Analysis

The simulation results and analysis of traffic load optimization problems in two scenarios are presented in this section, and the parameters of the simulation are shown in Table 2. Four situations are considered for relay: the number of the DRS *M* is equal to 1 (single DRS relay) and 4, 6 and 8 (multi-DRS relay). Based on the characteristics of two different scenarios, two scenarios are distinguished in terms of available bandwidth, maximum transmitting power, distance from SN and DRSs, communication time, relay period and parameters setting for ISL. The packet arrival process of SN and PS follows independent Poisson distribution.

Since capacity optimization is a common objective for wireless resource allocation, and the traffic load balancing for multi-DRS relay in SIN aims to obtain fairness allocation to avoid traffic congestion with acceptable system capacity, capacity performance and fairness performance are considered for analysis. Joint bandwidth and power allocation can improve the performance by resource sharing; hence, single resource allocation is introduced to be compared with the proposed schemes for two scenarios.

### 5.1. Simulation Results in Scenario 1

**Figure 4:**
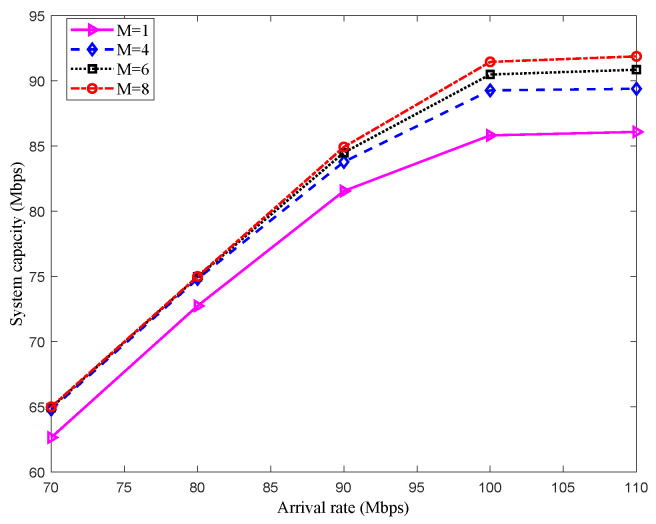
System capacity of different number of DRSs in Scenario 1.

The capacity of a multi-satellite relay system with different numbers of DRS is shown in Figure 4. The simulation adopts the Monte Carlo method, and the results are obtained by taking the average of 1000 times running. As shown in Figure 4, the system capacity increases with the increase of the arrival rate, but it can be seen from the slope of the curve that when the arrival rate of packets reaches a certain threshold, the system capacity’s increasing speed is slowed down. This is because the system capacity is gradually reaching its limit: that is, the ability of packets to be sent approximating the capacity boundary of SN during the visible time. At the same time, by comparing the performance of single DRS relay and multi-DRS relay, it can be found that the multi-DRS relay can improve the system capacity; then, boundary of the system capacity can be enhanced by adding the number of DRS, which is because the cooperative resource sharing among multiple DRSs can effectively enhance the utility of resource. Under low arrival rates (arrival rates are lower than 80 Mbit/s), the capacity change is not obvious by adding the number of DRS. This is because the relatively free state of the system stays on, and each of the arrived data packets can be served with reasonable resources. On the other hand, under high arrival rates (arrival rates are higher than 80 Mbit/s), the enhancement of the capacity is slow by adding DRSs; this is because that the SN only has one laser antenna, and the visible time is limited.

**Figure 5:**
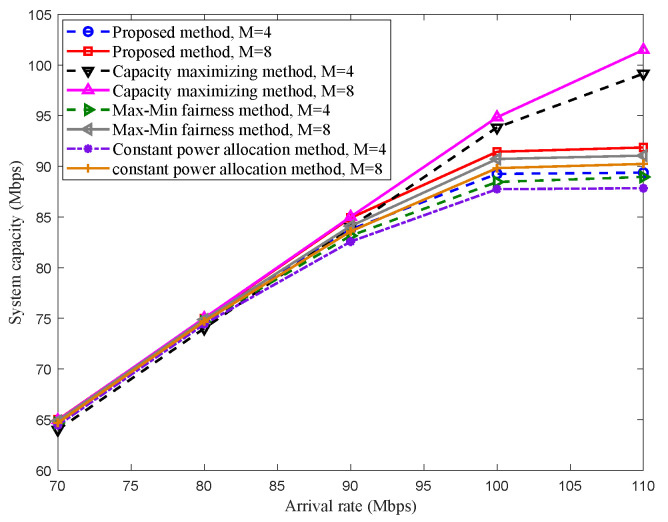
System capacity of different methods in Scenario 1.

Figure 5 shows the system capacity performance of different methods. The simulation adopts the Monte Carlo method, and the results are obtained by taking the average of 1000 times running. Three typical methods are considered for comparing with the proposed scheme, which are the capacity maximizing method [29], the Max–Min fairness method [30], and the constant power allocation (CPA) method, respectively. The first two methods use corresponding objective functions in the references as the optimization functions. Meanwhile, the CPA method is expressed as: under the optimization objective in this paper, the maximum transmitting power $P_{1,\mathrm{total}}$ is adopted for each DRS, and only the time-slot is optimized. It can be seen from Figure 5 that the boundary of the system capacity can be enhanced by adding DRS, which is similar to what is shown in Figure 4. Under low arrival rates (arrival rates are lower than 90 Mbit/s), the performance of the proposed scheme is almost the same with the capacity maximizing method. Under high arrival rates (arrival rates are higher than 90 Mbit/s), the performance of the proposed scheme is lower than the capacity maximizing method, while it is higher than other methods. It is because the proposed scheme optimizes the traffic load of multi-DRS with an acceptable loss of capacity based on PF criterion, and it allows multi-DRS with various channel conditions for different traffic loads, which improves the capacity performance compared with the Max–Min method. Furthermore, joint bandwidth and power optimization can enhance the capacity boundary ulteriorly.

**Figure 6:**
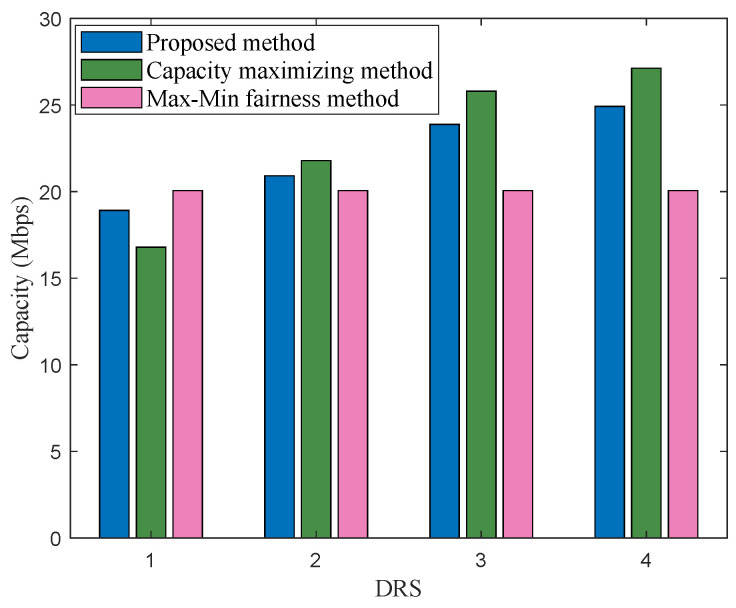
Capacity distribution for 4 DRSs in scenario 1.

The traffic load distribution performance among multi-DRS for one optimization is shown in Figure 6, where the number of DRSs M=4, and the arrival rate is 90 Mbit/s. As we can see, the capacity maximizing method can improve the system capacity by allocating resource preferentially to DRSs with better channel condition, which results in a wide variation in the distribution of capacity on the four DRSs. When the system reaches higher data arrival rates, the capacity maximizing method results in a high traffic load for ISLs with better channel conditions, which could cause data overload and congestion. The Max–Min fairness method lets each DRS obtain the same capacity performance; however, as it is shown in Figure 5, this traffic load-balancing method comes at the expense of a lot of capacity, which would greatly reduce the number of data packets that can be relayed in the limited visible time. Comparing with the above two methods, the proposed scheme can balance the traffic load among multi-DRS to some extent and prevent an overload of traffic for each DRS on the premise of ensuring a reasonable system capacity and transmitting data as much as possible.

### 5.2. Simulation Results in Scenario 2

**Figure 7:**
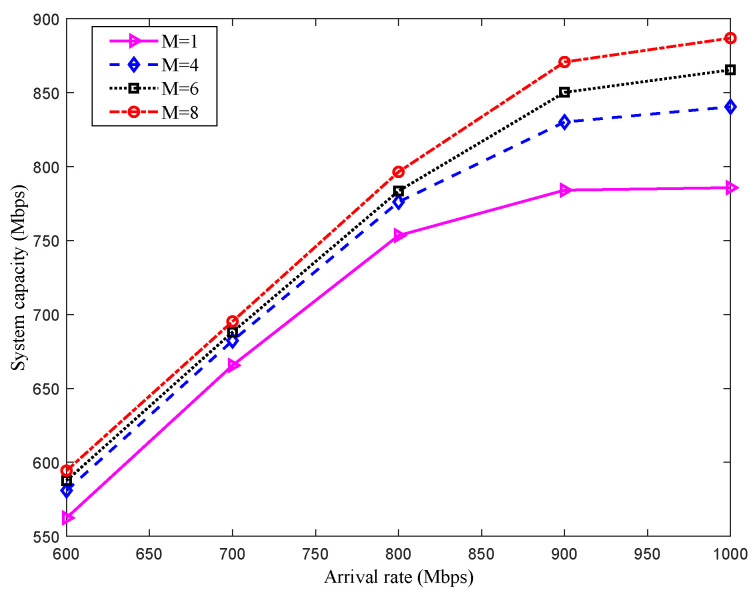
System capacity of different number of DRSs in scenario 2.

The system capacity performance with different numbers of DRS is shown in Figure 7. The simulation adopts the Monte Carlo method, and the results are obtained by taking the average of 1000 times running. Similar to scenario 1, the system capacity increases with the increase of the arrival rate, and when the arrival rate of packets reaches a certain threshold, the system capacity increasing speed is slowed down, and the multi-DRS relay can improve the system capacity. Under low arrival rates (arrival rates are lower than 700 Mbit/s), the capacity change is not obvious by adding the number of DRS. However, unlike scenario 1, under high arrival rates (arrival rates are higher than 700 Mbit/s), the capacity performance is enhanced markedly using multi-DRS relay. It is becausethe PS has multiple laser antenna, which can realize cooperative transmission with multi-DRS with power optimization and bandwidth sharing.

**Figure 8:**
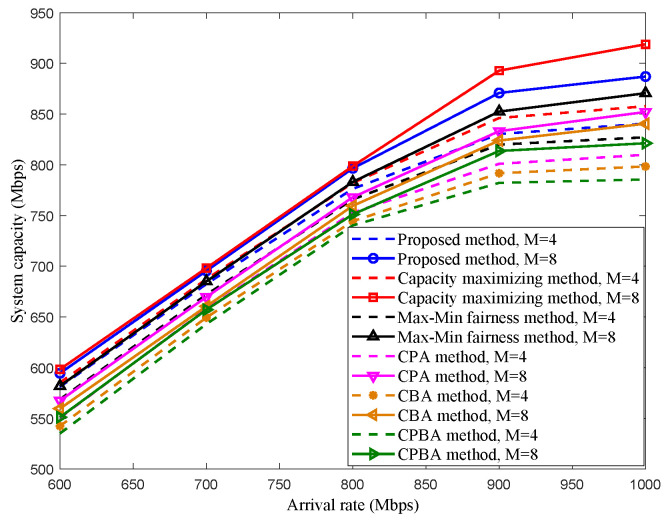
System capacity of different methods in scenario 2.

The system capacity performance of different optimization methods for scenario 2 is shown in Figure 8. The simulation adopts the Monte Carlo method, and the results are obtained by taking the average of 1000 times running. Five typical methods are considered to be compared with the proposed scheme, which are the capacity maximizing method [29], Max–Min fairness method [30], CPA method, constant bandwidth allocation (CBA) method, and constant power and bandwidth allocation (CPBA) method, respectively. The CPA method is expressed as: under the optimization objective in this paper, the total power is allocated equally to each DRS, which is equal to $P_{2,\mathrm{total}}/M$, and only the bandwidth allocation is optimized. Meanwhile, CBA means that the total bandwidth is allocated equally to each DRS, and only the power is optimized. Then, the CPBA method lets each DRS obtain an equal bandwidth and power solutions; thus, the bandwidth and power allocated to each DRS are B/M and $P_{2,\mathrm{total}}/M$, respectively. Similar to scenario 1, the capacity boundary can be enhanced by adding the number of DRS. The performances of the proposed method and the capacity maximizing method are almost the same under low arrival rates (arrival rates are lower than 800 Mbit/s), and the performance of the proposed scheme begins to degrade after the arrival rate is above 800 Mbit/s compared with the capacity maximizing method. On the other hand, the performance of the proposed scheme is significantly better than that of other methods except for the capacity maximizing methods in all arrival rate cases. This is because the joint optimization of wireless resources for *M* DRSs in each time-slot can improve the resource utility. In addition, the performance of the constant power and bandwidth allocation method, the constant bandwidth allocation method, the constant power allocation method and the proposed method increase successively, which proves that for the traffic load optimization problem of multi-satellite relay systems considered in this paper, the impact of bandwidth on system capacity is greater than that of power. Meanwhile, the combined optimization of bandwidth and power can further improve system capacity compared with the single dimensional resource optimization.

**Figure 9:**
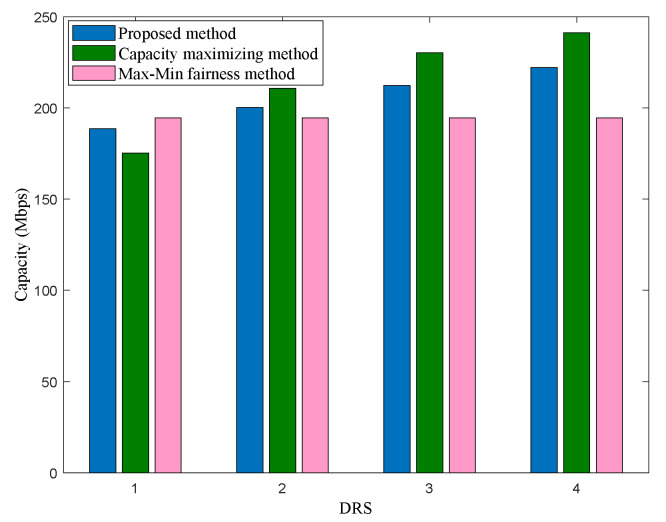
Capacity distribution for 4 DRSs in scenario 2.

The traffic load distribution performance among four DRSs for one optimization is shown in Figure 9. Similar to scenario 1, the capacity maximizing method allocates more limited resources to DRSs with better ISL conditions to improve the system capacity, resulting in a large difference in the performance distribution of four DRS. In this way, it is easy to lead to an overload of DRSs with better ISL conditions, which would cause congestion. The Max–Min fairness method is at the cost of sacrificing more system capacity, so that each DRS can obtain the same capacity and achieve the optimal balance of traffic load among four DRSs (with the best fairness), but it cannot guarantee that the data packets to be forwarded by PS can be relayed in the shortest possible time. The proposed scheme can effectively balance the traffic load of multi-DRS on the premise of ensuring a reasonable system capacity so as to avoid the congestion of some DRSs and long relay delay.

## 6. Conclusions

In this paper, the SIN structure model and the traffic load optimization problem of multi-DRS cooperative relay systems in SIN are studied. According to the definition of SIN, SIN based on DSC is represented as a DSCN model, and its main characteristics are analyzed. On this basis, a hybrid resource management architecture with central-distribution combination is designed to adapt to the multi-latitude, hierarchical and distributed radio resource management under the DSCN model. Based on the DSCN model, the mathematical models of two kinds of relay scenarios in SIN are given, and the traffic load optimization problems in two scenarios are proposed according to the PF criterion. Based on the convex optimization theory, it is proved that the two optimization problems proposed in this paper are convex optimization problems, and the closed-form solutions of the two problems in their dual domain are solved by dual transformation. Finally, according to the proposed hybrid resource management architecture, two iterative algorithms based on the subgradient method are designed to find the optimal solutions of the two problems in this paper.

Through simulation experiments and analysis, the accuracy of theoretical analysis and derivation in this paper are verified, and some inspiring conclusions are drawn as follows. (a) Multi-DRS cooperative relay can effectively improve system capacity compared with single DRS relay. (b) The improvement of cooperative relay capacity is constrained by the total radio resource, the signal transmission capability of SN and the mobility between the SN and DRSs, and enhancing the communication load capacity of the SN (such as increasing the number of laser antennas) can improve the relay performance. (c) The optimal allocation of bandwidth resource has more influence on the system capacity improvement than that of the power resource, and the combined optimization of power and bandwidth can effectively improve the upper bound of system capacity. (d) The capacity maximizing method improves the system capacity at the expense of traffic load distribution balance among multi-DRS, while the Max–Min fairness method enables DRSs to obtain the same traffic load but leads to a lower system capacity. Different from these methods, the schemes proposed in this paper can guarantee the system capacity at a reasonable level; at the same time, they can balance the traffic load well for multi-DRS with asymmetric channel conditions.

## Figures and Tables

**Table 1:** Comparison of existing works.

Classification	Representative Literature	Shortcomings
		Cannot be directly applied to the satellite
Terrestrial networks	[7,8,9,10,11,12]	cooperative relay system due to the difference
		between space links and terrestrial links
		Without considering the characteristics of the link
Satellite networks	[13,14,15,16,17,18,19]	between satellites and the resource optimization and
		traffic load balance for multi-DRS cooperative relay

**Table 2:** Simulation parameters for multi-DRSs relay in SIN.

Names of Parameters	Symbols	Values
Number of DRS	*M*	1, 4, 6 and 8
Bandwidth of SN in scenario 1	$B_{1}$	10 MHz
Bandwidth of PS in scenario 2	*B*	100 MHz
Distance between SN and DRSs in scenario 1	$d_{1,m}$	5000 km
Distance between PS and DRSs in scenario 2	$d_{2,m}$	5 km
Maximum transmitting power in scenario 1	$P_{1,\mathrm{total}}$	50 dBm
Maximum transmitting power in scenario 2	$P_{2,\mathrm{total}}$	100 dBm
Communication time in scenario 1	$T_{a}$	20
Relay period in scenario 2	$T_{b}$	40
Power ratio of LoS signal and scattering signal in scenario 1	$s_{1}^{2}/\sigma_{1}^{2}$	7 dB
Power sum of LoS signal and scattering signal in scenario 1	$s_{1}^{2}+\sigma_{1}^{2}$	8 dB
Power ratio of LoS signal and scattering signal in scenario 2	$s_{2}^{2}/\sigma_{2}^{2}$	8 dB
Power sum of LoS signal and scattering signal in scenario 2	$s_{2}^{2}+\sigma_{2}^{2}$	9 dB
Path fading coefficient in scenario 1	$\gamma_{1}$	2.5
Path fading coefficient in scenario 2	$\gamma_{2}$	2
AWGN power for ISL	$N_{0}$	$10^{-10}$
Iteration termination index	ε	0.01

## Data Availability

Not applicable.

## Abbreviations

The following abbreviations are used in this manuscript:

SIN	Space information network
DRS	Date relay satellite
DSC	Distributed satellite cluster
GEO	Geostationary earth orbit
MEO	Medium earth orbit
LEO	Low earth orbit
QoS	Quality of service
PF	Proportional fairness
ICL	Inter-cluster link
ISL	Inter-satellite link
PS	Primary satellite
PS	Primary satellite
DSCN	Distributed satellite cluster network
NCC	Network control center
DN	Destination node
SN	Source node
AWGN	Additive white Gaussian noise
LoS	Line of sight
SNR	Signal to noise ratio
CPA	Constant power allocation
CBA	Constant bandwidth allocation
CPBA	Constant power and bandwidth allocation
